# Property Comparison of Alkali-Activated Carbon Steel Slag (CSS) and Stainless Steel Slag (SSS) and Role of Blast Furnace Slag (BFS) Chemical Composition

**DOI:** 10.3390/ma12203307

**Published:** 2019-10-11

**Authors:** Jinyan Liu, Cheng Yi, Hongguang Zhu, Hongqiang Ma

**Affiliations:** 1 School of Mechanics and Civil Engineering, China University of Mining & Technology (Beijing), Beijing 100083, China; 2 Department of Civil Engineering, Shanxi University, Taiyuan 030006, China; 3 Department of Civil and Natural Resources Engineering, University of Canterbury, Christchurch 8041, New Zealand

**Keywords:** alkali activation, carbon steel slag, stainless steel slag, compressive strength, microstructural studies

## Abstract

In order to compare the properties of alkali-activated carbon steel slag (CSS) and stainless steel slag (SSS), the effects of sodium hydroxide/sodium silicate solution mass ratio (NH/NS), liquid/solid ratio and blast furnace slag (BFS) dosage on the compressive strength, hydration products and hydration degree of CSS and SSS were studied. Furthermore, a combination of X-ray diffraction (XRD), thermo-gravimetric analysis coupled with differential thermal analysis (TGA-DTA), Fourier transform infrared spectroscopy (FT-IR) and scanning electron microscope-energy dispersive spectrometer (SEM-EDS) were used to characterize the morphology and structure of alkali-activated CSS-BFS and SSS-BFS cementitious materials. As the results revealed, the primary hydrate of alkali-activated CSS and SSS is C-(A)-S-H with Q^2^ [SiO_4_] units, which has a low Ca/Si ratio and includes inert phases like a CaO-FeO-MnO-MgO solid solution (RO) in CSS while cuspidine, magnesiochromite etc. in SSS. More active C_3_S and β-C_2_S promote the alkali activation of CSS, whereas the less active γ-C_2_S hinders the depolymerization of SSS. The incorporation of BFS does not change the hydrate, whose seed effect is helpful for accelerating the depolymerization and polycondensation of CSS and SSS, especially for SSS, and makes the hydrate increase significantly. Owing to the high SiO_2_ and Al_2_O_3_ contents of SSS, the C-(A)-S-H chain length is increased, thus facilitating the polycondensation effect. In this study, the optimal NH/NS of CSS and SSS is NH/NS= 1:2, and the optimal liquid/solid ratio is 0.29. Compared to CSS–BFS, the C-(A)-S-H gel produced by SSS–BFS has lower Ca/Si and Al/Si ratios. Unlike CSS, pure SSS is inappropriate as an alkali-activated precursor and needs to be co-activated with BFS.

## 1. Introduction

With the continuous development of global steel industry in recent years, bulk solid wastes like steel slag and blast furnace slag (BFS) have tended to grow. Taking the two major types of steel slag, carbon steel slag (CSS) and stainless steel slag (SSS) [1,2,3], as an example, according to the latest data released by the World Steel Association (WSA) [4] and the International Stainless Steel Forum (ISSF) [5], the global crude steel production was 1808 million t in 2018, whereas stainless steel production was 50.7 million t, showing increases by 4.6% and 5.51%, respectively, over the same period of the previous year. Assuming that the CSS accounted for 15–20% of crude steel production [6] and that 1 t of SSS was produced for every 3 t of stainless-steel production [7], then the global productions of CSS and SSS exceeded 250 and 16 million t, respectively, in 2018. Besides, large amounts of BFS are produced annually. In 2017, the global pig iron production was 1180 million t. Assuming that the BFS production was 30% of pig iron production, then the BFS production could reach 390 million t. Today, BFS is widely used in the production of cement and concrete, while steel slag receives little utilization because of its low activity [8,9,10]. Currently, steel slag is used mostly for low value-added applications, including asphalt concrete aggregates, fillers for foundation engineering, supplementary cementitious materials, etc. [1,2,11,12,13,14,15]. This necessitates substantial piling up of steel slag, which causes environmental pollution, arable land occupation and resource wastage. At present, researchers in various countries are committed to seeking effective ways to enhance steel slag and increase the utilization rate of steel slag.

The methods of steel slag activation mainly include thermal activation [16,17,18], mechanical activation [19,20], chemical activation [9,12,18,21] and steel slag reconstruction [22,23], of which alkali activation is undoubtedly the current hot topic. Alkali activated materials (AAMs) are a class of cementitious materials formed by reacting pozzolanic or latently hydraulic materials like fly ash (FA), blast furnace slag (BFS) and metakaolin with alkaline activators. They have received widespread scholarly attention owing to their prominent physical properties such as high strength, high corrosion resistance, fire resistance, hazardous waste sealability, as well as the advantage of efficient industrial residue utilization [12,24,25,26,27]. On the other hand, alkali-activated materials have large drying shrinkage and poor toughness, which can cause cracking of paste, mortar or concrete, thus restricting their development [28,29,30,31].

Clearly, the use of steel slag as the precursor for preparing the alkali-activated steel slag-based cementitious material not only can improve the utilization rate of steel slag, but can also lower the cement content, which is favorable for energy conservation and environmental protection. Hu et al. [32] prepared geopolymer repair material through alkaline activation by adding 20% CSS into metakaolin and found that the material possessed a good compressive strength and an apparently better bonding strength with old concrete than the cement-based repair materials. Wang and Yan [10] observed that the BOF slag activating effect of NaOH was evident in the earlier hydration stage, which weakened in the later stage. The hydration products of BOF slag mainly included C-S-H containing Al, Mg, Fe, as well as Ca (OH)_2_. Gonzalez et al.’s [23] high-temperature reconstruction study based on Si, Al material addition into BOF slag revealed that low modulus alkali was enough to stimulate the activity of modified BOF slag, and that the hydration product was amorphous C (F,N)-A-S-H. Peng et al. [33] verified that the alkali activation could not fully stimulate the activity of pure CSS, while a combination of CSS and BFS was conducive to the development of cementitious system hydration. Thermal plus alkali activation was performed by Salman et al. [18] to endow the stainless-steel refining slag with cementing activity. Their results demonstrated that the activation process was accompanied by the decline of C_2_S and bredigite, and the final hydration product was C-S-H. Shi [9] showed that water glass can stimulate the cementitious activity of ladle slag at room temperature by adding BFS. The fabrication of steel slag and BFS-based cement-free cementitious materials by Cui et al. [34] and Tsai et al. [35] suggested that CSS and BFS could mutually facilitate hydration.

Overall, as a high-calcium precursor, the alkali-activated steel slag is less studied than the alkali-activated BFS system. The complex activation via multiple modes, or the combination of steel slag with other solid waste materials as the precursor for alkali activation are the major research directions [33,36,37]. On the other hand, the majority of existing works have focused on CSS. Indeed, the differences in the chemical composition, mineral phase and crystal structure among steel slag types lead to varying alkali activation properties. Further revelation is needed as to the alkali excitation mechanism of SSS. In view of the wide application of BFS in geopolymers, and the precedent of combining CSS with BFS as an alkali-activation precursor [9,33], this paper comparatively investigates the effects of sodium hydroxide/sodium silicate (NH:NS) solution mass ratio, liquid/solid ratio and BFS dosage on the polymer strength, non-evaporable water content, hydrates and the microstructure of CSS and SSS by using a mixture of NaOH and NS as an activator. While contributing to the further understanding of CSS and SSS as precursors for alkali activation, this also facilitates a deeper probing into the mechanism of BFS powder modification.

## 2. Materials and Methods

### 2.1. Materials

**Figure 1:**
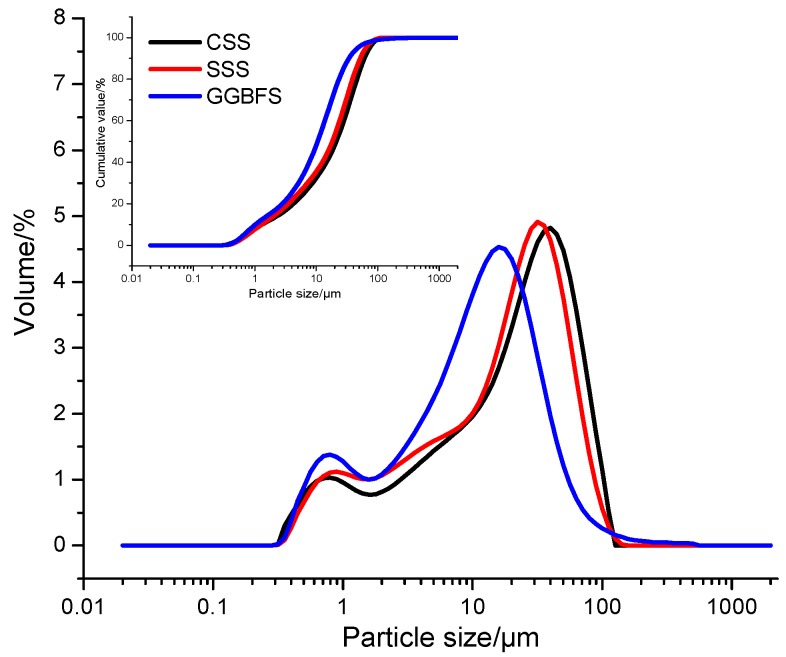
Particle size distribution of carbon steel slag (CSS)/stainless steel slag (SSS)/blast furnace slag (BFS).

The CSS and SSS were from Taiyuan Iron & Steel (Group) Co., Ltd. in Shanxi, China. The P.O 42.5 cement conforming to Chinese National Standard GB175-2007 were from Jigang Cement Co., Ltd. in Wenshui, Shanxi, China. In Figure 1, the particle size distributions of the tailings obtained through crushing, grinding, magnetic separation and sieving are presented. The median particle size d50 values of CSS, SSS and BFS were 21.141 μm, 18.220 μm and 10.529 μm, respectively. The alkali activators used were 96% analytical grade NaOH and sodium silicate solution having a modulus of 3.22 (26.5% SiO_2_, 8.5% Na_2_O, 65% H_2_O).

**Figure 2:**
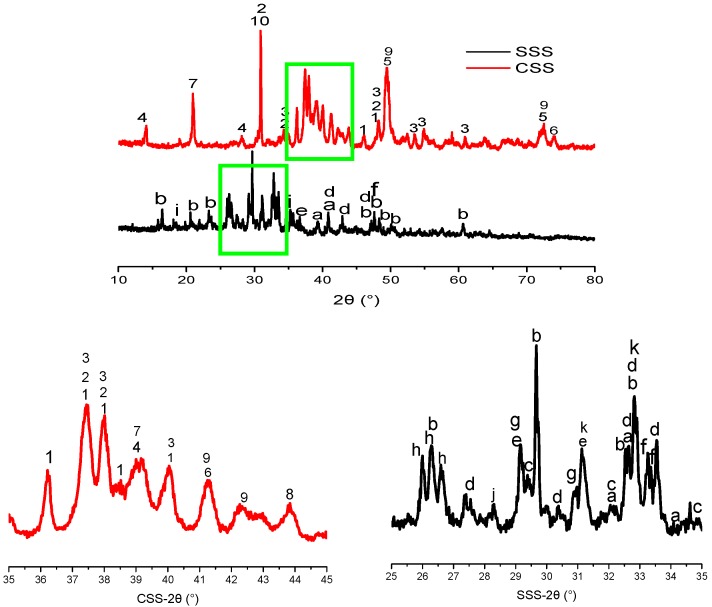
X-ray diffraction of CSS and SSS.

X-ray fluorescence (XRF) spectroscopy (XRF-1800, SHIMADZU, Kyoto, Japan) was used to analyze the chemical compositions of CSS, SSS and BFS (Table 1). X-ray diffraction (XRD, Bruker D8 ADVANCE, Bruker, Leipzig, Germany) was used to perform the phase analysis of CSS and SSS (Figure 2). Table 2 lists the details of phases. As can be seen, the main mineral components of CSS are Larnite (β-C_2_S), γ-C_2_S, C_3_S, Srebrodolskite, Wustite, Magnetite, Mayenite, CaO, RO, SiO_2_, etc. The main mineral components of SSS are γ-C_2_S, Larnite (β-C_2_S), C_3_S, Bredigite, Akermanite, Merwinite, Cuspidine, SiO_2_, Magnesiochromite (chromium spinels), Fluorite, Rankinite, etc. The cementitious activity of steel slag comes from C_3_S and C_2_S. The content of C_3_S is very small; while the main mineral phase C_2_S has two different crystalline forms, β-C_2_S and γ-C_2_S, in CSS and SSS.

### 2.2. Mixes Design and Specimens Preparation

To investigate the effects of BFS dosage, NH:NS, liquid-solid ratio on the alkali-activated CSS and SSS, the Portland cement paste samples of P.O 42.5 were used as the control group and a total of 22 set of samples were prepared by setting three BFS dosages (0%, 25%, 50%), four NH:NS (1:1, 1:1.5, 1:2, 1:2.5) and six liquid-solid ratios (0.27, 0.29, 0.31, 0.33, 0.35, 0.37). Table 3 lists the specific mix proportions.

After preparing 10 mol/L NaOH solution with water and solid NH, the alkali activator solutions were then prepared by adjusting the proportion of sodium silicate solution and NaOH solution, which were finally added with water to obtain the liquid-solid ratios as shown in Table 3. After uniform stirring, the solutions were allowed to cool to room temperature for 24 h [38,39]. For the preparation of the sample blocks, the activator solutions were added to CSS or SSS, stirred in a cement paste mixer for 2 min, and then poured into steel molds sized 40 mm × 40 mm × 40 mm [20,38]. The molds were vibrated on an electric vibrator table for 40 s to remove the air inside the samples, and then covered with polyester films and cured for 1 d in the T = 20 ± 1 °C, RH = 95 ± 1% conditions. After demolding, the samples were further cured in the aforementioned conditions. At curing ages of 3 d, 7 d, 28 d and 90 d, the unconfined compressive strength was examined. For the samples under tests of non-evaporable water content, XRD, TG-DTG (Thermogravimetry-Derivative Thermogravimetry), FT-IR and SEM-EDS, the net paste blocks needed to be cored, crushed and placed in acetone for 24 h to terminate hydration prior to the tests. Finally, after taking out and drying in an 80 °C to a constant weight [40], the samples were ground and sieved through a 0.07 mm mesh.

### 2.3. Testing Methods

#### 2.3.1. Unconfined Compressive Strength Test

The TYE-300 compression testing machine (Jianyi Instrument & Machinery Co., Ltd., Wuxi, China) was used to test the unconfined compressive strengths of the samples at 1 d, 3 d, 7 d, 14 d, 28 d and 90 d at a loading rate of 2.4 KN/s. The final strength value was obtained from the average of three samples of the same condition [38,39].

#### 2.3.2. Non-Evaporable Water Content Test

The non-evaporable water content, also known as the bound water content, was obtained by dividing the mass difference between the samples at 105 °C and 1000 °C by the mass at 105 °C. The specific test procedure and calculation method were the same as those used by Wang et al [20] and Yi et al [38].

#### 2.3.3. Phase Analysis

D8 ADVANCE X-ray diffractometer (Bruker, Leipzig, Germany) was used to perform the phase analysis. The SSS series samples were tested with the copper target, while the CSS series samples were tested with the cobalt target. The reason for selecting the cobalt target was that the CSS samples contained Fe element, which would generate strong fluorescent X-rays after being provoked. As a result, diffraction backgrounds would be superposed, which was detrimental to the analysis. 2θ range: 10–80°; scan rate: 6 °/min; and scan step size: 0.02.

#### 2.3.4. TG-DTG Analysis

STA 449 F3 thermal analyzer (Netzsch, Bavaria, Germany) was used to perform the TG-DTG analysis. During the test, the temperature was raised from room temperature to 900 °C at a 10 °C/min rate under a nitrogen atmosphere.

#### 2.3.5. FT-IR Analysis

The functional groups of the sample compounds were characterized with Nicolet iS10 FTIR spectrometer (Madison, WI, USA) for further analysis of the structural composition. The spectral range was set to 400–4000 cm^−1^; the resolution was set as 0.09 cm^−1^; and the dynamic adjustment 130,000 times/s.

#### 2.3.6. Microstructure Analysis

JSM-7001F thermal field emission SEM (JEOL, Toky, Japan) was utilized to observe the sample morphology at an accelerating voltage of 5 KV. Meanwhile, QX200 energy-dispersive spectrometer (Bruker, Germany) was used for direct qualitative and semi-quantitative observations of trace elements in the specific micro-regions.

## 3. Results and Discussion

### 3.1. Unconfined Compressive Strength

**Figure 3:**
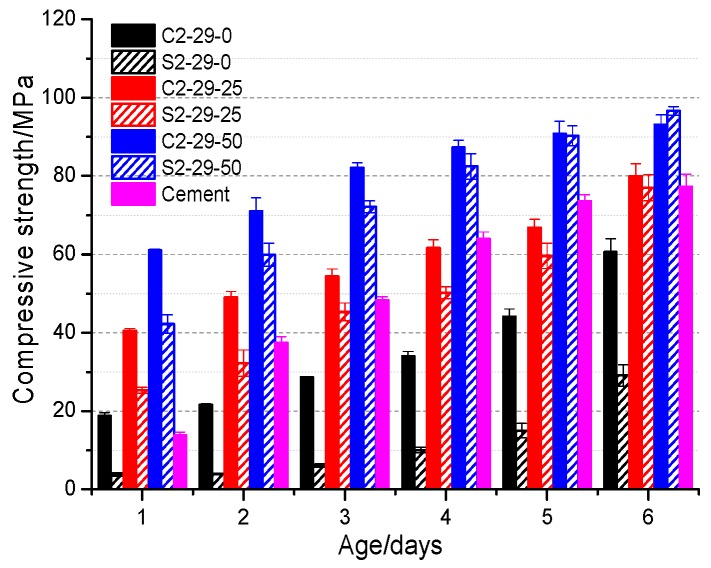
Strength development of CSS and SSS with different BFS dosage.

In Figure 3, the compressive strengths of alkali-activated steel slag with different BFS contents are displayed. As is clear, both the CSS and SSS specimens exhibit enhancing compressive strength with increasing age and BFS dosage. Compared to the P.O 42.5 cement specimen, the alkali-activated steel slag–BFS specimens have higher early strengths. To be specific, the 1 d, 3 d and 7 d strengths of C2-29-25 are all higher than the cement specimen of the same age, while for S2-29-25, only the 1 d strength is greater than the cement specimen. C2-29-50 and S2-29-50 can attain higher early compressive strengths, and their 90 d compressive strength are higher than the P.O 42.5 cement. The SSS specimens (especially pure SSS specimens) show significantly lower compressive strength than the CSS specimens. Increasing BFS helps to prominently narrow the strength gap between the two specimen types. This gap also decreases with extending age. For instance, the compressive strength of S2-29-50 approaches C2-29-50 on 28 d and then surpasses C2-29-50 on 90 d. Compared to CSS, the compressive strength of SSS specimens grows at significantly faster rates. As an example, S3-29-0’s 3 d, 7 d, 14 d, 28 d and 90 d strength growth rates are 2.89%, 55.50%, 65.30%, 49.45% and 93.68%, respectively, which are markedly higher than C2-29-0’s 15.21%, 32.09%, 20.34%, 28.06% and 37.04%. The incorporation of BFS contributes more to the strength growth rate of SSS specimens. Taking S2-29-25 specimen as an example, its 1 d, 3 d, 7 d, 14 d, 28 d and 90 d strength growth rates are 566.71%, 723.40%, 645.39%, 399.20%, 296.84% and 164.61%, respectively, which are significantly higher than C2-29-25’s 114.39%, 125.30%, 89.14%, 78.42%, 51.08% and 31.66%. In summary, pure CSS is more suitable than pure SSS as an alkali-activated precursor. The BFS incorporation increases the strength of alkali-activated CSS and SSS, and the effect of BFS on SSS is more significant.

**Figure 4:**
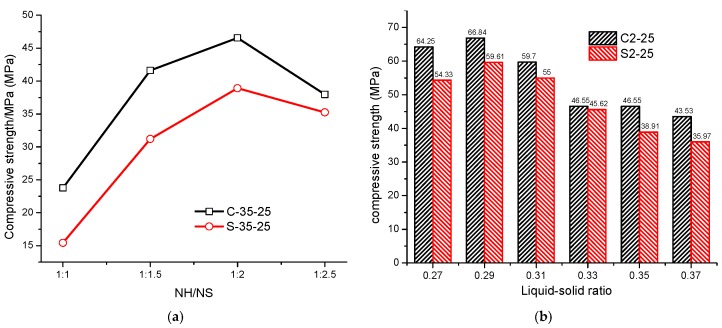
Compressive strength of CSS and SSS: (**a**) different sodium hydroxide/sodium silicate (NH/NS) (28 d); (**b**) different liquid-solid ratio (28 d).

Figure 4a presents the compressive strengths of alkali-activated CSS and SSS at various sodium hydroxide/sodium silicate (NH/NS) mass ratios. As is clear, at an age of 28 d, both CSS and SSS reach maximum strengths when the NH/NS ratio is 1:2. The reason is as follows: The reaction process of alkali-activated cementitious materials comprises the dissolution, depolymerization and polycondensation of precursor [41]. Low NaOH (NH/NS = 1:2.5) is detrimental to the precursor dissolution, which delays or decelerates the depolymerization and polycondensation. Macroscopically, this is manifested as the decrease of compressive strength. The increase of NaOH (NH/NS = 1:2) accelerates the precursor dissolution and depolymerization. Moreover, Na_2_SiO_3_ provides an adequate amount of [SiO_4_]^4−^ to accelerate the polycondensation reaction, thereby leading to increased strength. The excessive NaOH (NH/NS ≥ 1:1.5) leads to a hyper fast reaction, where the limited hydrates accumulate rapidly on the surface of partial precursor particles to hinder the continuation of the reaction. As a result, there is a limited increase in strength.

In Figure 4b, the compressive strengths of alkali-activated CSS and SSS are illustrated at various liquid/solid ratios. As is clear, the CSS and SSS reach maximum compressive strengths on 28 d when the liquid/solid ratio is 0.29. At higher liquid/solid ratios (>0.29), large pores are formed inside the alkali-activated specimens, which go against the strength growth [38]. Lower liquid/solid ratios lead to higher density. However, excessively low liquid/solid ratios (<0.27) are detrimental to slurry mixing, so that the precursor and the activator reacted insufficiently. This, in turn, affects the formation of the polymer skeleton to result in decreased strength. Given the high density and low water demand of steel slag studied herein, the optimal liquid/solid ratio is lower than the study by Yi et al [38].

### 3.2. Non-Evaporable Water 

**Figure 5:**
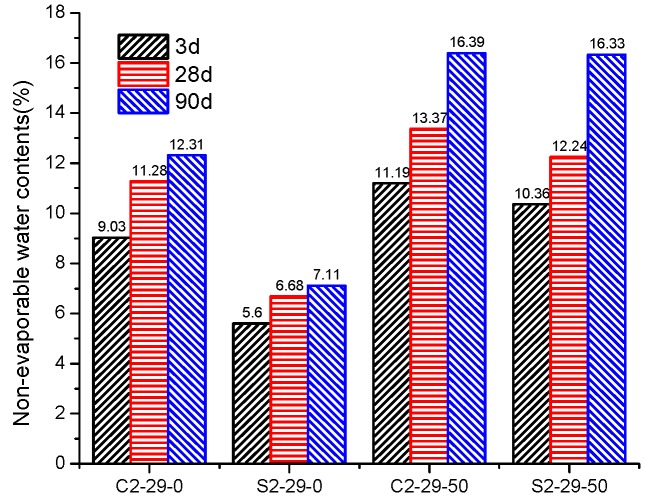
Non-evaporable water contents of CSS and SSS with different BFS dosage.

**Figure 6:**
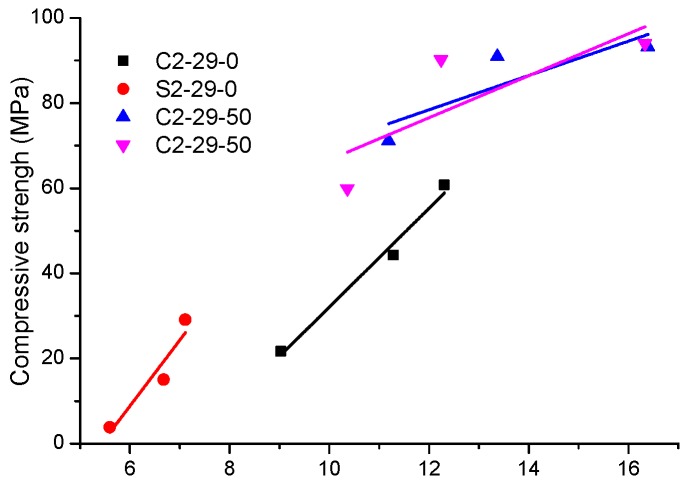
Relationship between non-evaporable water content and compressive strength of pastes for 3 d, 28 d and 90 d.

Non-evaporable water is also known as bound water. The higher content of bound water implies greater total production of alkaline aluminosilicate hydrate [38]. From Figure 5, it can be seen that CSS contains more non-evaporable water than SSS. The incorporation of BFS is conducive to producing more alkaline aluminosilicate, which allows the quantity of SSS hydrate to increase significantly to approximate that of CSS hydrate. This is consistent with the strength growth trends (Figure 3) of S2-29-50 and C2-29-50. An approximately linear relationship has been found between the non-evaporated water contents and the developed compressive strength of paste (Figure 6). As a consequence of the alkaline activation reaction progress, more hydrates are formed and more strength is developed. Thus, the compressive strength increased with the non-evaporated water contents.

### 3.3. XRD Analysis

**Figure 7:**
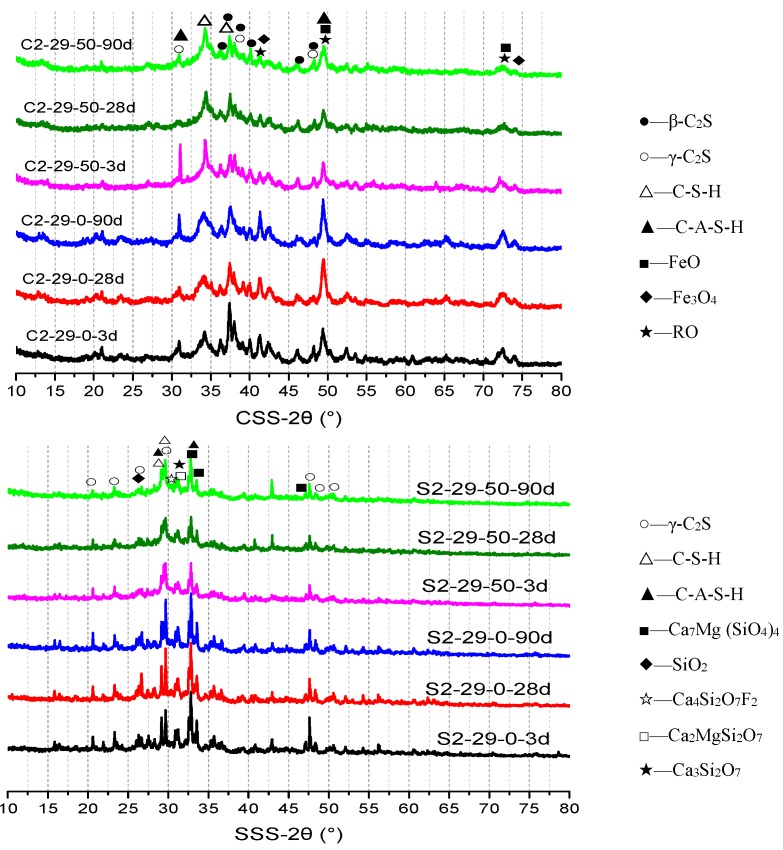
XRD analysis of CSS and SSS specimens at different ages.

Figure 7 displays the XRD diffraction patterns of alkali-activated CSS and SSS specimens at different ages. As can be seen, due to the rapid hydration of C_3_S, the C-S-H and C-A-S-H peaks already appear for the pure CSS specimen at an age of 3 d, which is accompanied by the disappearance of C_3_S peak. β-C_2_S and γ-C_2_S react slowly and continuously, whose peaks decay gradually with increasing age but still exist on 90 d. The BFS incorporation promotes the alkali activation, which is beneficial to the formation of well-crystallized C-S-H and C-A-S-H. This is manifested by markedly weakened β-C_2_S and γ-C_2_S peaks, as well as sharp and heightened peaks of C-S-H and C-A-S-H. No obvious changes are observed in the peaks of RO phase (Mg_1−x_Fe_x_O), wustite (FeO) or magnetite (Fe_3_O_4_), suggesting that these phases do not participate in the alkali activation reaction.

During alkali activation of the pure SSS specimen, γ-C_2_S and bredigite (Ca_7_Mg (SiO_4_)_4_) decrease slightly with age [18]. There are a few hydrates, or the hydrates are amorphous or semi-crystalline, which are hardly recognizable even via the 27.5°–37.5° hump [12,38]. On 90 d, a weak C-S-H peak appears at 29.1°. The enhancement of the hump and C-S-H peak after BFS incorporation confirms that the SSS needs to be combined with BFS, in order to be alkali-activated at room temperature. Despite overlaps with the peak position of bredigite (Ca_7_Mg (SiO_4_)_4_), the increases in hydrates C-A-S-H and C-S-H with age remain observable via XRD. This phenomenon is more apparent within a 28–90 d range following the incorporation of BFS. The increased hydrates ensure a steady growth of SSS specimens’ later strength (Figure 3). Alkali activation produces no significant effects on cuspidine (Ca_4_Si_2_O_7_F_2_), magnesiochromite (Mg-Cr_2_O_4_) or fluorite (CaF_2_) in SSS (Table 2). Similar to CSS, the compressive strength of the SSS specimens is derived entirely from the hydrates C-S-H and C-A-S-H.

### 3.4. TG-DTG Analysis

**Figure 8:**
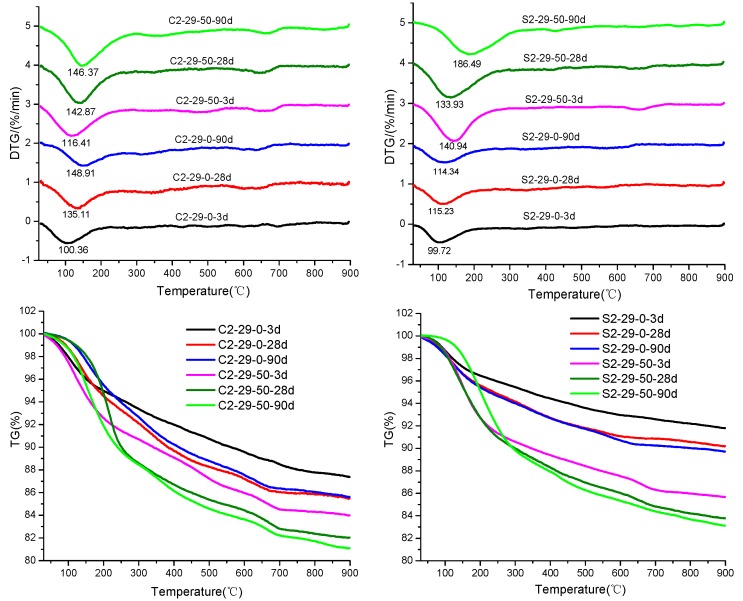
TG-DTG analysis of CSS and SSS with different BFS dosage (0% and 50%) for 3, 28, and 90 d.

The TG-DTG analysis is extensively used in the monitoring of the hydration process and the quantitative determination of C-S-H, Ca (OH)_2_ and other hydrates [38,42]. In this study, no presence of Ca (OH)_2_ is noted at 450–500 °C, showing consistency with the XRD findings [18,40]. Figure 8 presents the TG-DTG analysis results of C2-29-0, C2-29-50, S2-29-0 and S2-29-50 on 3 d, 28 d and 90 d. In the DTG spectrum, the primary endothermic peak is located between 100–180 °C, which represents the dehydration of C-A-S-H or C-S-H gel [43]. Another weak peak appearing near 650 °C corresponds to the CaCO_3_ decomposition as detected by FI-RT. At temperatures above 700 °C, the weight remains essentially unchanged. Due to the poor crystallization of fine particles formed by carbonization [43] and the presence of Na+, the decomposition temperature of CaCO_3_ is lower than its natural range (700–800 °C).

In Table 4, the mass losses of C2-29-0, C2-29-50, S2-29-0 and S2-29-50 specimens at 35–300 °C are listed, as well as their total mass losses. As is clear, the mass losses increase with the increasing BFS content and age. The SSS specimens exhibit less mass losses than the CSS specimens. This difference between the two is reduced after the BFS incorporation, showing consistency with the strength growth trend (Figure 3) and the non-evaporable water content (Figure 6) results. As the quantity and density of C-(A)-S-H gels increases, the mass loss of dehydration endothermic peak also increases, which is manifested macroscopically as the enhancement of compressive strength. Obviously, the SSS specimens exhibit higher gel growth rates.

There are the following two typical trends from the peak temperature perspective: The BFS-free CSS specimens have higher peak temperatures than the corresponding SSS specimens of the same ages, especially on 28 d and 90 d. However, the peak temperature of the SSS specimen with 50% BFS rises more significantly. As an example, the S2-29-50 specimen has a 63.1% (72.15 °C) higher peak temperature than the S2-29-0 on 90 d, which reaches a maximum value. The second trend is that the peak temperatures of the same specimen types increase with age [44]. The increase in peak temperature indicates a tighter bond between C-(A)-S-H gel and its bound water, a higher degree of polymerization, or the formation of smaller pores for the gel itself [44,45]. For the SSS specimens, the gel compressive strength increases continuously (Figure 3).

### 3.5. FT-IR analysis

**Figure 9:**
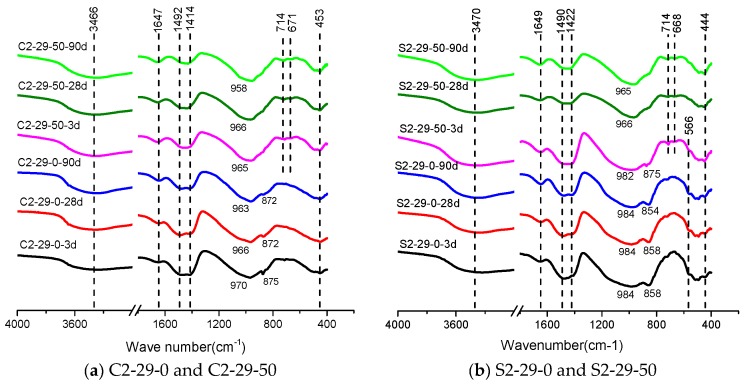
TF-IR spectra of pastes with different BFS dosage (0% and 50%) after 3, 28 and 90 days of curing. (**a**) C2-29-0 and C2-29-50; (**b**) S-2-29-0 and S2-29-50.

The phase transitions (including molecular and molecular bond structures) of alkali-activated CSS and SSS can be studied by FT-IR [38,46]. Figure 9 shows the FT-IR spectra (spectral range = 400–4000 cm^−1^) of C2-29-0, C2-29-50, S2-29-0, S2-29-50 at different ages.

The apparent broad band in the range of 2800 cm^−1^ to 3700 cm^−1^ is caused by the stretching vibration of the OH group in H_2_O or hydroxides [12,47]. This band should be from the bound water in C-(A)-S-H since no hydroxide is detected in TGA or XRD [18]. At 1647 cm^−1^, a band resulting from the bending vibration of H-O-H in the H_2_O molecules is found, which is also attributed to the bound water contained in C-(A)-S-H. The peak intensities of the above two bands are enhanced with increasing age and BFS dosage [38,44], indicating that the product C-(A)-S-H of alkali-activated CSS and SSS grows with age [17,18], and that the incorporation of BFS is conducive to the production of more C-(A)-S-H. The absorption peak at 1420 cm^−1^~1500 cm^−1^ corresponds to the asymmetric vibration of O-C-O, suggesting carbonization of the samples [18,48]. In addition, the position of 450 cm^−1^~570 cm^−1^ corresponds to the bending vibration of O-Si-O.

The large, broad main band near 970 cm^−1^ is attributed to the asymmetric stretching of the Q^2^ [SiO_4_] tetrahedra’s Si-O bond [12,47], which is important evidence for the alkali-activated polymerization process [38]. Its peak frequency variation depends on the alkali-activated precursor material and the properties of hydrates. Unlike pure CSS, the alkali-activated pure SSS has a rather high peak frequency, which is always at 980 cm^−1^ within 90 d. This not only demonstrates that the Si-O bond in SSS possesses stronger crosslinking capacity and bond energy, but also suggests that the alkali activation produces little effect on pure SSS. This agrees with the result of markedly lower compressive strength of the alkali-activated pure SSS (Figure 3). BFS can promote the depolymerization of [SiO_4_] tetrahedron in the precursor, so that the peak position of main band shifts towards the low wave number. The larger shift range of SSS indicates that BFS has a stronger depolymerization promoting effect on SSS, thus facilitating subsequent polycondensation. BFS’s promotion effect on SSS is unobvious at 3 d and persistent after 28 d, which corresponds to the continuous later strength growth of SSS (Figure 3).

The weak peak between 930 cm^−1^ and 815 cm^−1^ is related to γ-C_2_S [18]. In CSS, this peak appears between 871 cm^−1^ and 875 cm^−1^, while in SSS, it appears in the vicinity of 857 cm^−1^, showing a slight deviation from the typical peak position (860 cm^−1^) [18]. This is probably attributable to the existence of Mn, Cr and other elements in the crystal. As shown in Figure 9a, the peak weakens gradually with age, reflecting the progressive reaction process of C_2_S, and disappears after the incorporation into BFS, implying that slag can promote fast depolymerization of CSS. However, the depolymerization effect of BFS on SSS precursors was not obvious at 3 d (Figure 9b). It is not until 28 d and 90 d that the peak eventually disappears.

Both CSS and SSS show new bands at 670 cm^−1^ after the BFS addition, which correspond to the symmetric stretching vibration of Si-O-Si (Al) bond at the junction of [SiO_4_] tetrahedrons and [AlO_4_] tetrahedrons [34]. This represents the polycondensation of silicon (aluminum) oxygen tetrahedrons. In other words, the addition of BFS facilitates the polycondensation of a cementitious system, whose macroscopic manifestation is the improvement of strength.

### 3.6. SEM-EDS Analysis

**Figure 10:**
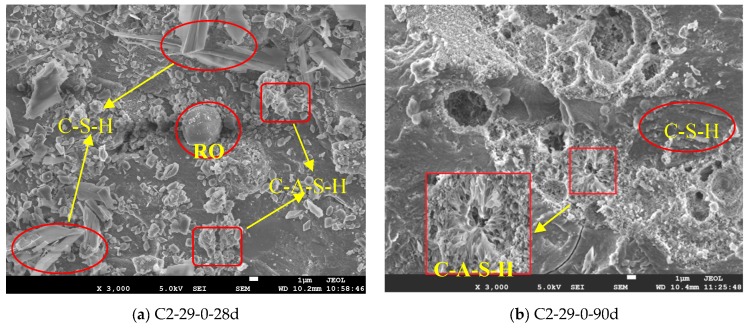

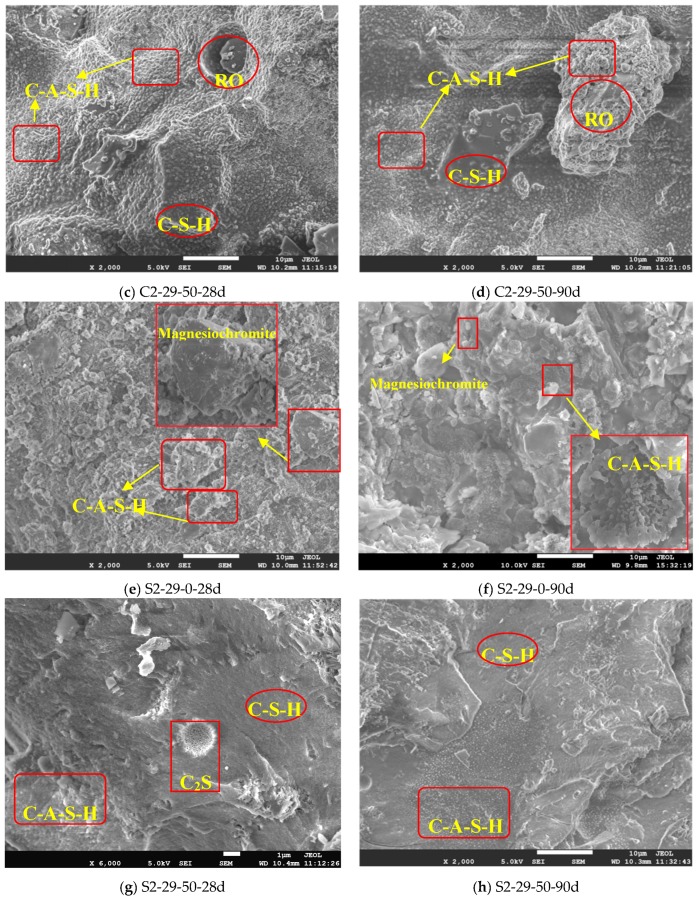
SEM images of alkali-activated CSS and SSS with different BFS dosages (0% and 50%) at 28 d and 90 d. (**a**) C2-29-0-28d; (**b**) C2-29-0-90d; (**c**) C2-29-50-28d; (**d**) C2-29-50-90d; (**e**) S2-29-0-28d; (**f**) S2-29-0-90d; (**g**) S2-29-50-28d; (**h**) S2-29-50-90d.

The cross-sectional morphologies of CSS and SSS samples are analyzed based on the SEM images, and an elemental analysis is performed by EDS. The results are displayed in Figure 10. At 28 d, the alkali-activated hydration products of C2-29-0 are platy, columnar or scattered C-S-H, as well as flocculated C-A-S-H. As shown in Figure 10a, the two hydration products are limited in quantity and distributed independently, with slightly poor integrality. At 90 d, C-S-H and C-A-S-H are each extended and mingled together. Most pores are filled inward by the clustered C-A-S-H via the pore wall to produce a dense effect (Figure 10b), so that the strength is enhanced (Figure 3).

Notably, S2-29-0 only produces small amounts of independent C-A-S-H at 28 d. Meanwhile, the remaining small particles are distributed in disorderly layers, which are formed by the sodium silicate bonding of non-polymerized raw materials (Figure 10e). As a result, the macroscopic strength becomes considerably low (Figure 3). At 90 d, C-A-S-H exhibits an increased quantity and flower cluster-like spread instead of a layered pattern, whose integrality though remains poor (Figure 10f). After adding BFS, C-(A)-S-H of C2-29-50 and S2-29-50 increased significantly, resulting in a remarkable increase in the sample compactness and integrality, which is in line with the trends of macroscopic strength development (Figure 3). The villous reaction ring peripheral to C_2_S particles in Figure 10g reflects the progressive reaction process of C_2_S, which also guarantees the continuous growth of the SSS sample strength in the later stage.

Both CSS and SSS contain inert phases, namely the RO phase and magnesiochromite. The RO phase has coarse grains and a smooth surface. After the addition of BFS, the RO phase surface is entangled by C-(A)-S-H only after going through a long period of time (Figure 10d), which is a weak link of the gel structure. Meanwhile, magnesiochromite has a smooth and flat surface (Figure 10e). The magnesiochromite encapsulation performance of hydrate C-(A)-S-H (Figure 10f) reflects its Cr sealing effect, a harmful heavy metal element.

The 90 d C-(A)-S-H gels of C2-29-0, C2-29-50, S2-29-0, S2-29-50 and P.O 42.5 specimens were subjected to EDS analysis by sampling 30 points per specimen. Table 5 lists the average results. As can be seen, the steel slag hydrate C-(A)-S-H gel has a smaller Ca/Si ratio and a larger Al/Si ratio than the P.O 42.5 hydrate C-S-H. At the same BFS dosage, both the Ca/Si and Al/Si ratios of the SSS specimens are lower than CSS. After the incorporation of BFS, Ca/Si increases, whereas Al/Si decreases for both types of specimens. According to the chemical composition data of raw materials (Table 1), SSS has higher SiO_2_, slightly higher Al_2_O_3_ and lower CaO contents than CSS, which is the primary reason for its low Ca/Si and Al/Si ratios. After participation in the alkali activation reaction, BFS releases its own Ca and Al while exerting a remarkable depolymerization effect on the steel slag to cause a substantial release of Si. Assuming that most of Ca and Al in the BFS raw material can be released, the final polycondensation product will have increased Ca/Si and decreased Al/Si since BFS contains significantly more CaO than Al_2_O_3_.

## 4. Reaction Mechanism

Steel slag is a Ca–Al–Si material, which undergoes high temperature and rapid cooling processes during formation and contains substantial crystals and metastable vitreous bodies. By the action of an alkaline activator, the thermodynamically unstable vitreous bodies depolymerize and recombine to form a dense slurry structure. The characteristics of alkali-activated CSS-BFS and SSS-BFS cementitious materials are influenced greatly by not only by the BFS content, NH/NS and liquid/solid ratio, but also by the mineral phase, crystallization degree and chemical composition.

The leading component of steel slag studied herein is C_2_S, while the C_3_S content is rare, thus leading to its considerably low activity. C_2_S has various crystal structures, which correspond to β-C_2_S in CSS and γ-C_2_S in SSS. The activity difference between the two originates mainly from the Ca-O bond, while the Si-O bond difference is tiny [49]. The hydration activity of γ-C_2_S is inferior to β-C_2_S partially because there is only one regular octahedral form of [CaO_6_] in γ-C_2_S unlike the two different coordination states of [CaO_6_] and [CaO_8_] in β-C_2_S, thus forming a more stable crystal structure. The inferior hydration activity is also attributed to the absence of active oxygen atoms with high charge density in γ-C_2_S [50]. 

SSS has higher SiO_2_, slightly higher Al_2_O_3_ and lower CaO contents than CSS, and the oxide composition between SSS and BFS is closer, but pure SSS exhibits weaker alkali activation properties because of its higher crystallization degree (Figure 2 and Figure 9).

As an excellent precursor [25,41], the activity of BFS is linked to the remote disordered vitreous structure. The vitreous structure consists of a continuous Ca-rich phase with a rather large internal specific surface area and a dense discontinuous Si-rich phase. The Si-O bond energy is approximately three times higher than that of Ca-O bond. During alkali activation, the high concentration OH^−^ first overcomes the decomposition and activation energies of Ca-rich phase to let it dissolve and expose the Si-rich phase. The reaction of the Si-rich phase occurs subsequently, but persistently and slowly [51]. Ca^2+^ in the glass phase increases the disorderliness of structure, which acts as a network corrector to distort and depolymerize the network structure, thereby reducing the polymerization degree of raw material [41]. The BFS incorporation allows the production of more hydrates (Figure 8). It also facilitates the depolymerization of γ-C_2_S and shifts the asymmetric stretching of Q^2^ tetrahedra’s Si-O bond towards a low wave number, which is accompanied by peak intensity decay of γ-C_2_S (Figure 9).

Similar to the alkali-activated BFS, the primary hydrate of alkali-activated steel slag is amorphous C-(A)-S-H gel in a Q^2^ [SiO_4_] tetrahedral form. The addition of Al^3+^ elongates the chain length of C-(A)-S-H [52,53,54], which correspond to the shift of the peak position near 970 cm^−1^ towards a low wave number direction and the formation of a new absorption peak near 670 cm^−1^ (Figure 9). The C-(A)-S-H after Al substitution is uniform (Figure 10) and dense with high strength (Figure 3).

The strength of alkali-activated steel slag cementitious system comes from the hydrate C-(A)-S-H, and Si-rich raw materials are conducive to the production of more C-(A)-S-H hydrate. Unlike CSS, the Si-O bond of alkali-activated pure SSS shows unobvious depolymerization due to the high γ-C_2_S content and the doping of elements like Mn and Cr, thus resulting in a limited polycondensation effect and low strength. After the incorporation of BFS, on the one hand, C-(A)-S-H is formed by depolymerization and recombination of BFS to enhance the strength. On the other hand, porous C-(A)-S-H with large specific surface area acts as a “seed” [55,56], adsorbing free water, increasing OH- concentration in the system, promoting depolymerization and improving the pore structure. As a result, the band near 980 cm^−1^ in SSS shifts significantly towards the low wave number (Figure 9). Once depolymerized, the higher Si content of SSS than CSS ensures the growth of C-(A)-S-H to allow the continuous strength enhancement. 

The alkali activation process is accompanied by a decrease in the active products (Figure 7), such as C_3_S, β-C_2_S in CSS and γ-C_2_S, bredigite in SSS. However, the inert phases, such as the RO phase (Mg_1−x_Fe_x_O), wustite (FeO), magnetite (Fe_3_O_4_) in CSS and cuspidine (Ca_4_Si_2_O_7_F_2_), magnesiochromite (Mg-Cr_2_O_4_), fluorite (CaF_2_) in SSS, do not participate in the reaction (SEM image) (Figure 10). To some extent, the presence of these inert phases forms a weak area of slurry, thereby weakening the alkali activation effect.

## 5. Conclusions

In this paper, the hydration properties of alkali-activated CSS/SSS–BFS cementitious materials are studied. The BFS content, NH/NS and liquid/solid ratio all affect the strength and hydration progression of the studied materials. The following conclusions are drawn:

(1) The quantity of C-(A)-S-H, the primary hydrate of alkali-activated steel slag cementitious materials, determines the materials’ hydration properties, such as the strength and non-evaporable water content. C-(A)-S-H increases with the increasing BFS content. For CSS and SSS, the optimal alkali dosage is NH/NS = 1:2, which can balance the depolymerization and polycondensation, and the optimal liquid/solid ratio is 0.29, which ensures the formation of the gel skeleton and the compactness of paste.

(2) The contents of steel slag’s mineral components β-C_2_S, γ-C_2_S and chemical components, including CaO, SiO_2_ and Al_2_O_3,_ determine their alkali activation performance as a precursor. SSS is not easily alkali-activated due to the stable crystal structure of γ-C_2_S, which is reflected in the delay or even termination of depolymerization. Nevertheless, its high Al_2_O_3_ and SiO_2_ contents elevate its potential as a precursor. Without doubt, depolymerization is important for steel slag, especially for SSS.

(3) BFS’s beneficial seed effect on the alkali activation of steel slag is reflected in two aspects: The production of C-(A)-S-H through the alkali activation and the promotion of steel slag depolymerization. The increase in the BFS content is helpful for producing C-(A)-S-H gel with high Ca/Si and low Al/Si ratios, thus improving the gel density. Compared with CSS, SSS has higher SiO_2_, slightly higher Al_2_O_3_, and lower CaO, resulting in SSS-BFS gel with lower Ca/Si and Al/Si ratios than CSS-BFS gel.

(4) The inert phases in the steel slag like RO and magnesiochromite do not participate in the alkali activation reaction. In the gel system, they are encapsulated by C-(A)-S-H to form a weak link. This proves, from another perspective, that the alkali-activated steel slag possesses sealing properties against harmful metals like Cr.

(5) Considering the effect and cost of alkali activation, pure CSS can be used as a precursor, while SSS needs to be activated jointly with BFS.

## Figures and Tables

**Table 1:** Oxide composition of cementitious materials by XRF analysis (wt%).

Materials	CaO	SiO_2_	Al_2_O_3_	M_g_O	Fe_2_O_3_	SO_3_	Cr_2_O_3_	MnO	T_i2_O
CSS	41.36	19.79	9.78	4.02	20.73	0.47	0.55	1.06	0.77
SSS	39.90	34.19	12.30	2.28	4.70	0.64	1.88	0.55	1.30
BFS	38.44	30.58	14.04	10.57	0.35	2.36	—	0.57	1.93
Cement	62.09	20.88	5.57	2.43	2.40	5.02	—	—	0.31

**Table 2:** Mineral phases of CSS and SSS.

CSS			SSS		
No.	Mineral Phase	Chemical Formula	No.	Mineral Phase	Chemical Formula
1	Larnite (β-C_2_S)	Ca_2_SiO_4_	a	Larnite (β-C_2_S)	Ca_2_SiO_4_
2	γ-C_2_S	Ca_2_SiO_4_	b	γ-C_2_S	Ca_2_SiO_4_
3	C_3_S	Ca_3_SiO_5_	c	C_3_S	Ca_3_SiO_5_
4	Srebrodolskite	Ca_2_Fe_2_O_5_	d	Bredigite	Ca_7_Mg (SiO_4_)_4_
5	Wustite	FeO	e	Akermanite	Ca_2_MgSi_2_O_7_
6	Magnetite	Fe_3_O_4_	f	Merwinite	Ca_3_Mg (SiO_4_)_2_
7	Mayenite	Ca_12_Al_14_O_33_	g	Cuspidine	Ca_4_Si_2_O_7_F_2_
8	Calcium Oxide	CaO	h	SiO_2_	SiO_2_
9	RO	Mg_1−x_Fe_x_O	i	Magnesiochromite (chromium spinels)	MgCr_2_O_4_
10	SiO_2_	SiO_2_	j	Fluorite	CaF_2_
			k	Rankinite	Ca_3_Si_2_O_7_

**Table 3:** Mix proportion of specimen’s paste.

Specimens	Liquid-Solid Ratio	NH:NS^a^	BFS/CSS^a^	Specimens	Liquid-Solid Ratio	NH:NS ^a^	BFS/CSS ^a^
C2-29-0	0.29	1:2	0:100	S2-29-0	0.29	1:2	0:100
C2-29-25	0.29	1:2	25:75	S2-29-25	0.29	1:2	25:75
C2-29-50	0.29	1:2	50:50	S2-29-50	0.29	1:2	50:50
C1-35-25	0.35	1:1	25:75	S1-35-25	0.35	1:1	25:75
C1.5-35-25	0.35	1:1.5	25:75	S1.5-35-25	0.35	1:1.5	25:75
C2-35-25	0.35	1:2	25:75	S2-35-25	0.35	1:2	25:75
C2.5-35-25	0.35	1:2.5	25:75	S2.5-35-25	0.35	1:2.5	25:75
C2-27-25	0.27	1:2	25:75	S2-27-25	0.27	1:2	25:75
C2-31-25	0.31	1:2	25:75	S2-31-25	0.31	1:2	25:75
C2-33-25	0.33	1:2	25:75	S2-33-25	0.33	1:2	25:75
C2-37-25	0.37	1:2	25:75	S2-37-25	0.37	1:2	25:75
OPC	0.29	-	-	-	-	-	-

^a^ mass ratio.

**Table 4:** Mass losses (%) for the pastes after 3, 28 and 90 days of curing at different temperature ranges (35–300 **°C** and total mass loss).

Specimen	Mass Losses (%)					
	3 Days		28 Days		90 Days	
	35–300 °C	Tatal	35–300 °C	Tatal	35–300 °C	Tatal
C2-29-0	6.51	12.50	7.93	14.54	7.39	14.42
S2-29-0	4.51	8.16	5.77	9.73	5.84	10.12
C2-29-50	9.15	15.85	11.45	17.97	11.49	18.83
S2-29-50	9.32	14.23	10.03	16.23	10.22	16.91

**Table 5:** The average of the atomic percentage of each element in the three samples (At%).

Specimen	Ca	Al	Si	Ca/Si	Al/Si
C2-29-0	10.55	4.74	8.37	1.260	0.566
C2-29-50	14.68	2.73	8.05	1.824	0.339
S2-29-0	12.82	3.02	11.61	1.104	0.260
S2-29-50	11.21	1.64	7.62	1.472	0.215
Cement	17.43	3.56	8.63	2.019	0.142
